# Ring-strain release in neutral and dicationic 7,8,17,18-tetra­bromo-5,10,15,20-tetra­phenyl­porphyrin: crystal structures of C_44_H_26_Br_4_N_4_ and C_44_H_28_Br_4_N_4_^2+^·2ClO_4_^−^·3CH_2_Cl_2_

**DOI:** 10.1107/S2056989016007349

**Published:** 2016-05-20

**Authors:** W. Robert Scheidt, Hugues F. Duval, Allen G. Oliver

**Affiliations:** a 235 Nieuwland Science Hall, Department of Chemistry and Biochemistry, University of Notre Dame, Notre Dame, IN 46556, USA

**Keywords:** crystal structure, prophyrin, hydrogen bonding, ring puckering

## Abstract

Two porphyrin complexes were studied to determine the effects of protonation on ring deformation within the porphyrin. The porphyrin 7,8,17,18-tetra­bromo-5,10,15,20-tetra­phenyl­porphyrin, C_44_H_26_Br_4_N_4_, was selected because the neutral species is readily doubly protonated to yield a dication, which was crystallized here with perchlorate counter-ions as a di­chloro­methane tris­olvate, C_44_H_28_Br_4_N_4_^2+^·2ClO_4_^−^·3CH_2_Cl_2_. The centrosymmetric neutral species is observed to have a mild ‘ruffling’ of the pyrrole rings and is essentially planar throughout; intra­molecular N—H⋯N hydrogen bonds occur. In contrast, the dication exhibits considerable deformation, with the pyrrole rings oriented well out of the plane of the porphyrin, resulting in a ‘saddle’ conformation of the ring. The charged species forms N—H⋯O hydrogen bonds to the perchlorate anions, which lie above and below the plane of the porphyrin ring. Distortions to the planarity of the pyrrole rings in both cases are very minor. The characterization of the neutral species represents a low-temperature redetermination of the previous room-temperature analyses [Zou *et al.* (1995 ▸). *Acta Cryst.* C**51**, 760–761; Rayati *et al.* (2008 ▸). *Polyhedron*, pp. 2285–2290], which showed disorder and physically unrealistic displacement parameters.

## Abstract 2

The different degrees of ring folding of a neutral and dicationic porphyrin are described and discussed.

## Chemical context   

Ring folding in porphyrins has long been of inter­est with characteristics such as ruffling, doming and saddling resulting in strain relief about the ring. In particular, the inter­actions within the constrained environment of the tetra-pyrrole core predominantly affect the orientation of the pyrrole rings. Two porphyrin mol­ecules were studied to examine the effects of protonation of the pyrrole nitro­gen atoms upon the overall geometry of the porphyrin ring systems. The porphyrin: 7,8,17,18-tetra­bromo-5,10,15,20-tetra­phenyl­porphyrin (I), H_2_TPPBr_4_ was adopted for this study. It readily accepts two protons forming a dicationic species (II), [H_4_TPPBr_4_]^2+^. The neutral porphyrin (I) has previously been reported in two different, room-temperature determinations (Zou *et al.*, 1995 ▸; Rayati *et al.*, 2008 ▸). However, those two structures display disorder that is not present in the low-temperature determination provided herein.

## Structural commentary   

**Figure 1:**
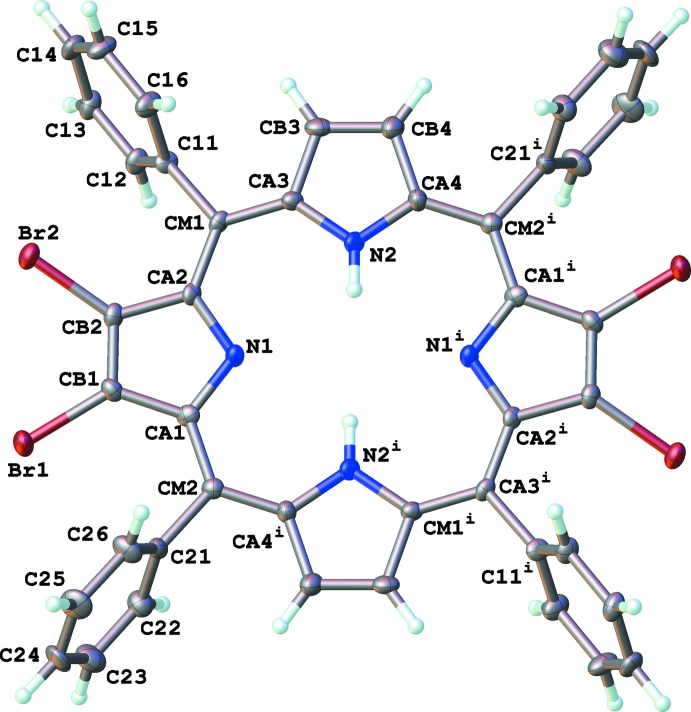
Structure and labeling scheme of (I). Atomic displacement parameters are depicted at 50% probability. H atoms are depicted as spheres of an arbitrary radius. [Symmetry code: (i) −*x*, −*y*, −*z*.]

**Figure 2:**
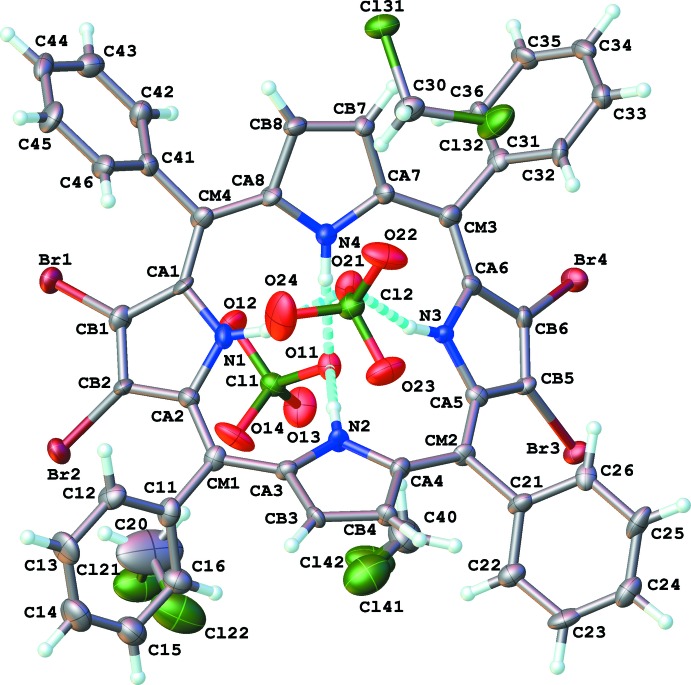
Structure and labelling scheme of (II). Atomic displacement parameters are depicted at 50% probability. H atoms are depicted as spheres of an arbitrary radius. Hydrogen bonds are represented as light-blue dashed lines.

The neutral porphyrin (I) was found to crystallize about the center of symmetry at the origin (Fig. 1 ▸). Distinctly different, the dicationic porphyrin (II) was found to crystallize with one complete porphyrin dication, two perchlorate ions and three mol­ecules of di­chloro­methane solvent of crystallization in the asymmetric unit (Fig. 2 ▸). Thus, the geometry of (I) is influenced by symmetry, while the geometry of (II) is independent of such constraints. In both studies, we elected to use the *meta*-carbon atoms of the porphyrin ring (labeled as CM*n* in the Figures; *n* = atom number) as the basis for an arbitrary mean plane for analyzing distortions.
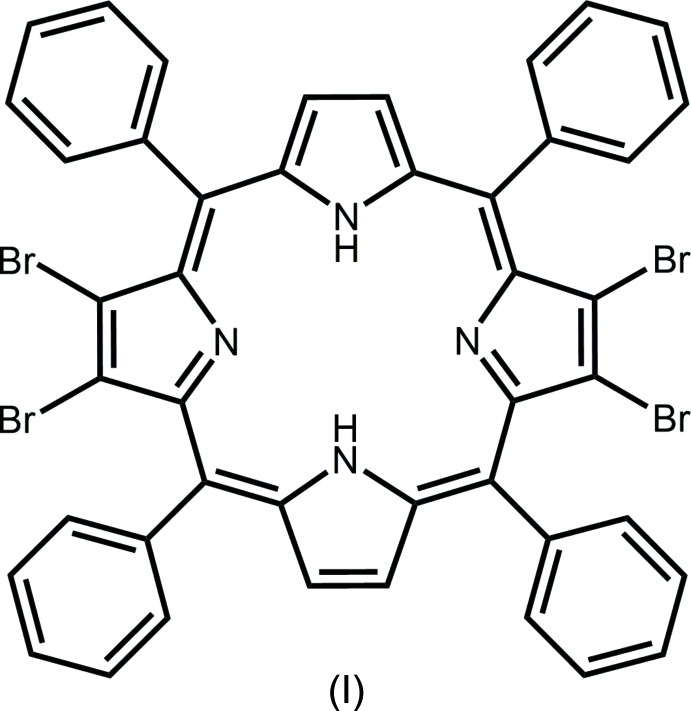
 
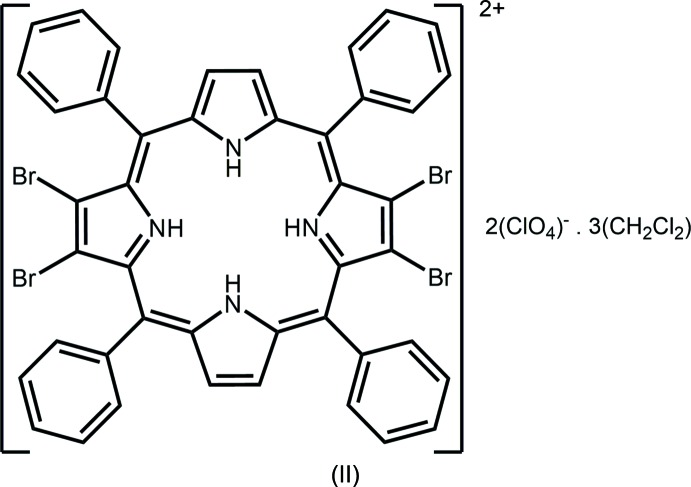


**Figure 3:**
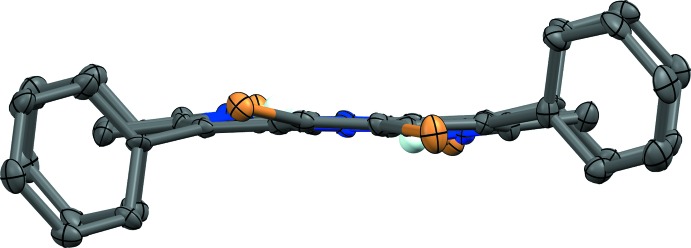
View through the porphyrin plane of (I) showing ring ‘ruffling’. H atoms, except pyrrole H atoms, have been omitted for clarity.

The neutral compound (I) exhibits very mild ‘ruffling’ of the pyrrole rings. The two independent pyrrole rings form periplanar angles of 3.0 (3) and 6.5 (3)° with the four porphyrin *meta*-carbon atoms (Table 1 ▸). This is largely influenced by the lack of steric hindrance of the two hydrogen atoms within the core of the porphyrin ring (Fig. 3 ▸). This lack of hindrance is also reflected in the intra­molecular N—H⋯N hydrogen bonds formed in the core that have typical *D*⋯*A* distances (Table 2 ▸). However, these intra­molecular hydrogen bonds are not well directed, as demonstrated by the relatively constrained N—H⋯N angles. The pyrrole rings experience very little distortion, with the greatest deviation from the mean-plane being −0.018 (3) Å for C*B*2 (Table 3 ▸). The ruffling of the ring is reflected more so in the deviations of the bromine and *ipso*-carbon atoms of the phenyl groups from the mean plane (Table 4 ▸). It should be noted that due to the center of symmetry, the transannular pairs of pyrrole rings are tilted in opposite directions with respect to the mean plane. Presumably this also plays a role in reducing steric hindrance of the pyrrole hydrogen atoms.

**Figure 4:**
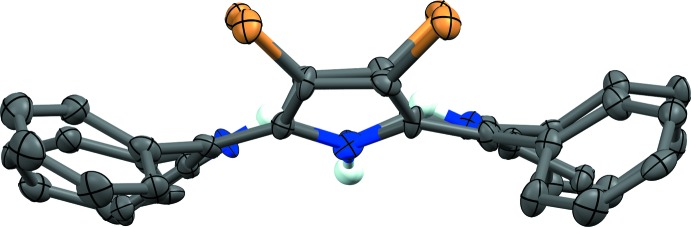
View through the porphyrin plane of (II) demonstrating ring ‘saddling’. H atoms, except pyrrole H atoms, have been omitted for clarity.

In contrast the dicationic porphyrin (II) relieves strain by adopting a ‘saddled’ conformation (Fig. 4 ▸). In this fashion, steric repulsion between the four hydrogen atoms intruding on the core of the porphyrin is significantly reduced. Furthermore, due to the presence of charge-balancing perchlorate anions, each pair of transannular pyrrole nitro­gen atoms form hydrogen bonds to one oxygen atom of either perchlorate anion (N1/N3⋯O21, N2/N4⋯O25, Fig. 2 ▸, Table 5 ▸).

Surprisingly, the pyrrole rings in (II) do not adopt any crystallographic symmetry. Crystallographically, each pair of rings oriented ‘up’ and ‘down’ (arbitrarily defined) form different angles with respect to the *meta*-carbon plane. Inspection of the structure shows that the bromo-pyrrole rings are inclined in the same fashion (we have arbitrarily defined this as ‘down’ or a negative periplanar angle with regards to the pyrrole nitro­gen atoms with respect to the porphyrin mean plane). In contrast with (I), the pyrrole rings in (II) form angles ±30° with respect to the mean porphyrin plane (Table 1 ▸). Compared with (I) wherein one bromine atom is deformed ‘above’ the pyrrole plane and the other ‘below’, the bromine atoms in (II) are all oriented out of the mean plane of their respective pyrrole rings in the same fashion (*i.e.* all of the deviations from the mean pyrrole plane are negative). The atoms of the pyrrole rings are essentially co-planar with the largest deviation from the mean plane for any pyrrole atom being −0.027 (7) Å for C*A*4 (Table 3 ▸).

Comparing bond distances around the neutral and dicationic porphyrin ring systems reveals little change in the bond distances associated with the pyrrole rings or backbone (see CIF files). Thus, in either a neutral or charged state the porphyrin consists largely of delocalized bonds, rather than the single-bond/double-bond formalism.

## Supra­molecular features   

The neutral compound (I) packs with typical van der Waals contacts. Potential close contacts from C16 to the pyrrole of an adjacent mol­ecule have the shortest heavy-atom contact around 3.45 Å.

In contrast, compound (II) is formed with hydrogen bonds from the pyrrole nitro­gen atoms to perchlorate oxygen atoms (Fig. 2 ▸, Table 5 ▸ for details). Remaining inter­molecular contacts throughout the structure are all usual van der Waals inter­actions.

## Database survey   

Inspection of the Cambridge Structure Database (Version 5.38 plus 1 update; Groom *et al.*, 2016 ▸) reveals three structures that incorporate the H_2_TPPBr_4_ moiety. Two structures (GOGNIA: Rayati *et al.*, 2008 ▸; LINPON: Zou *et al.*, 1995 ▸) are room-temperature determinations of the low-temperature structure (I) reported herein. Examination of those two structures reveals several underlying problems, such as disorder and unreasonable atomic displacement parameters that are not present in this study. The third compound that incorporates H_2_TPPBr_4_ is a co-crystallant with C60 fullerene (TUBPAJ: Karunanithi & Bhyrappa, 2015 ▸). To the best of our knowledge, the dicationic species (II) has not been structurally characterized in any form.

## Synthesis and crystallization   

Compound (I) was prepared following literature procedures (Callot, 1973 ▸; Crossley *et al.*, 1991 ▸). Compound (II) was prepared with procedures as previously described (Cheng *et al.*, 1997 ▸).

## Refinement details   

Crystal data, data collection and structure refinement details are summarized in Table 6 ▸. All non-hydrogen atoms were refined with anisotropic atomic displacement parameters. C-bound hydrogen atoms were included in geometrically calculated positions. N-bound hydrogen atoms were initially located from a difference Fourier map and subsequently included using a riding model. *U*_iso_(H) = 1.2*U*_eq_(C/N); C—H distances were set at 0.95 Å and N—H set at 0.88 Å for (I) and (II). Due to the age of the data and an infelicity in data archiving, only the printed structure-factor tables and final residuals file were available. Data were reconstituted from these tables into an *h k l F* σ(*F*) format file and the atomic models refined against these to result in the structures contained herein. It was not considered reasonable to attempt to resynthesize and recrystallize the compounds and collect new intensity data.

## Supplementary Material

Crystal structure: contains datablock(s) I, II, global. DOI: 10.1107/S2056989016007349/hb7580sup1.cif

Structure factors: contains datablock(s) II. DOI: 10.1107/S2056989016007349/hb7580IIsup3.hkl

CCDC references: 1477658, 1477657

Additional supporting information:  crystallographic information; 3D view; checkCIF report

## Figures and Tables

**Table 1:** Pyrrole periplanar angles (°) Angles with respect to the mean four atom *meta*-carbon plane. A ‘negative’ angle represents an arbitrary orientation with the pyrrole N atom below the mean porphyrin plane.

Pyrrole Ring	(I)	(II)
N1—C*A*1—C*B*1—C*B*2—C*A*2	3.0 (3)	31.0 (5)
N2—C*A*3—C*B*3—C*B*4—C*A*4	6.5 (3)	−30.1 (5)
N3—C*A*5—C*B*5—C*B*6—C*A*6		33.6 (4)
N4—C*A*7—C*B*7—C*B*8—C*A*8		−23.2 (3)

**Table 2:** Hydrogen-bond geometry (Å, °) for (I)

*D*—H⋯*A*	*D*—H	H⋯*A*	*D*⋯*A*	*D*—H⋯*A*
N2—H2⋯N1	0.88	2.47	2.973 (5)	117
N2—H2⋯N1^i^	0.88	2.40	2.921 (5)	118

Symmetry code: (i) 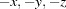.

**Table 3:** Deviations from pyrrole planes for (I) and (II) (Å)

Atom	(I)	(II)
N1	−0.008 (3)	−0.012 (7)
C*A*1	−0.004 (3)	0.006 (7)
C*A*2	0.015 (3)	0.013 (7)
C*B*1	0.014 (3)	0.002 (7)
C*B*2	−0.018 (3)	−0.009 (7)
Br1	−0.117 (8)	−0.28 (2)
Br2	0.403 (7)	−0.28 (2)
		
N2	0.006 (3)	0.021 (7)
C*A*3	−0.001 (3)	−0.006 (7)
C*A*4	−0.009 (3)	−0.027 (7)
C*B*3	−0.005 (3)	−0.011 (7)
C*B*4	0.008 (3)	0.023 (7)
		
N3		−0.015 (6)
C*A*5		0.017 (6)
C*A*6		0.007 (7)
C*B*5		−0.013 (7)
C*B*6		0.004 (7)
Br3		−0.283 (18)
Br4		−0.114 (19)
		
N4		0.005 (8)
C*A*7		0.000 (7)
C*A*8		−0.007 (8)
C*B*7		−0.004 (8)
C*B*8		0.006 (8)

**Table 4:** Deviations of peripheral atoms from mean *meta*-carbon plane for (I) and (II) (Å)

Atom	(I)	(II)
C11	−0.240 (7)	−0.038 (19)
C21	0.205 (8)	0.194 (18)
C31		0.061 (18)
C41		0.232 (19)

**Table 5:** Hydrogen-bond geometry (Å, °) for (II)

*D*—H⋯*A*	*D*—H	H⋯*A*	*D*⋯*A*	*D*—H⋯*A*
N1—H1⋯N2	0.88	2.57	3.018 (12)	113
N1—H1⋯O21	0.88	2.12	2.956 (14)	158
N2—H2⋯N1	0.88	2.60	3.018 (12)	110
N2—H2⋯N3	0.88	2.59	3.026 (12)	111
N2—H2⋯O11	0.88	2.07	2.896 (12)	157
N3—H3⋯O21	0.88	2.08	2.932 (13)	162
N4—H4⋯N3	0.88	2.62	3.034 (12)	110
N4—H4⋯O11	0.88	2.01	2.844 (13)	159

**Table 6:** Experimental details

	(I)	(II)
Crystal data		
Chemical formula	C_44_H_26_Br_4_N_4_	C_44_H_28_Br_4_N_4_^2+^·2ClO_4_^−^·3CH_2_Cl_2_
*M*_r_	930.33	1386.02
Crystal system, space group	Monoclinic, *P*2_1_/*n*	Monoclinic, *P* *n*
Temperature (K)	130	130
*a*, *b*, *c* (Å)	13.883 (3), 6.7448 (13), 19.110 (4)	12.903 (3), 13.761 (3), 14.876 (3)
β (°)	102.00 (3)	96.67 (3)
*V* (Å^3^)	1750.3 (7)	2623.5 (10)
*Z*	2	2
Radiation type	Mo *K*α	Mo *K*α
μ (mm^−1^)	4.64	3.53
Crystal size (mm)	0.15 × 0.10 × 0.05	0.33 × 0.17 × 0.06

Data collection		
Diffractometer	Enraf–Nonius fast area-detector	Enraf–Nonius fast area-detector
Absorption correction	Part of the refinement model (Δ*F*) (*DIFABS*; Walker & Stuart, 1983 ▸)	Part of the refinement model (Δ*F*) (*DIFABS*; Walker & Stuart, 1983 ▸)
*T*_min_, *T*_max_	0.72, 1.00	0.65, 1.00
No. of measured, independent and observed [*I* > 2σ(*I*)] reflections	4589, 4589, 3439	11251, 11251, 8745
(sin θ/λ)_max_ (Å^−1^)	0.701	0.703

Refinement		
*R*[*F* ^2^ > 2σ(*F* ^2^)], *wR*(*F* ^2^), *S*	0.059, 0.156, 1.08	0.060, 0.185, 1.06
No. of reflections	4589	11251
No. of parameters	235	640
No. of restraints	0	2
H-atom treatment	H-atom parameters constrained	H-atom parameters constrained
Δρ_max_, Δρ_min_ (e Å^−3^)	1.17, −1.41	1.03, −1.05
Absolute structure	–	Classical Flack method preferred over Parsons because s.u. lower (Flack, 1983 ▸)
Absolute structure parameter	–	−0.032 (14)

Computer programs: *MADNES* (Pflugrath & Messerschmidt, 1989 ▸), *SHELXS97* (Sheldrick, 2008 ▸), *SHELXL2014* (Sheldrick, 2015 ▸), *OLEX2* (Dolomanov *et al.*, 2009 ▸), *Mercury* (Macrae *et al.*, 2008 ▸) and *publCIF* (Westrip, 2010 ▸).

## supplementary crystallographic information

### Crystal data

**Table:**

C_44_H_28_Br_4_N_4_^2+^·2ClO_4_^−^·3CH_2_Cl_2_	*F*(000) = 1368
*M**_r_* = 1386.02	*D*_x_ = 1.755 Mg m^−^^3^
Monoclinic, *P**n*	Mo *K*α radiation, λ = 0.71073 Å
*a* = 12.903 (3) Å	Cell parameters from 250 reflections
*b* = 13.761 (3) Å	θ = 1.1–20.5°
*c* = 14.876 (3) Å	µ = 3.53 mm^−^^1^
β = 96.67 (3)°	*T* = 130 K
*V* = 2623.5 (10) Å^3^	Prism, blue-green
*Z* = 2	0.33 × 0.17 × 0.06 mm

### Data collection

**Table:**

Enraf–Nonius fast area-detector diffractometer	11251 measured reflections
Radiation source: ROTATING ANODE	11251 independent reflections
Graphite monochromator	8745 reflections with *I* > 2σ(*I*)
Detector resolution: 8.53 pixels mm^-1^	θ_max_ = 30.0°, θ_min_ = 2.5°
ELLIPSOID–MASK FITTING scans	*h* = −16→16
Absorption correction: part of the refinement model (Δ*F*) (*DIFABS*; Walker & Stuart, 1983)	*k* = 0→19
*T*_min_ = 0.65, *T*_max_ = 1.00	*l* = −19→18

### Refinement

**Table:**

Refinement on *F*^2^	Secondary atom site location: difference Fourier map
Least-squares matrix: full	Hydrogen site location: mixed
*R*[*F*^2^ > 2σ(*F*^2^)] = 0.060	H-atom parameters constrained
*wR*(*F*^2^) = 0.185	*w* = 1/[σ^2^(*F*_o_^2^) + (0.0886*P*)^2^ + 17.5195*P*] where *P* = (*F*_o_^2^ + 2*F*_c_^2^)/3
*S* = 1.06	(Δ/σ)_max_ < 0.001
11251 reflections	Δρ_max_ = 1.03 e Å^−^^3^
640 parameters	Δρ_min_ = −1.05 e Å^−^^3^
2 restraints	Absolute structure: Classical Flack method preferred over Parsons because s.u. lower (Flack, 1983)
Primary atom site location: structure-invariant direct methods	Absolute structure parameter: −0.032 (14)

### Special details

**Table:**

Experimental. Diffraction data were measured with an Enraf-Nonius FAST area detector to 55.56 deg in 2 theta. With the hardware and software supplied for the diffractometer, the data collection process provides substantial redundancy but not necessarily completion up to the limiting resolution. At a resolution of 0.83 Å (52 deg in 2 theta) essentially full coverage of data were met. Successful and suitable refinement of the structure supports this.
Geometry. All esds (except the esd in the dihedral angle between two l.s. planes) are estimated using the full covariance matrix. The cell esds are taken into account individually in the estimation of esds in distances, angles and torsion angles; correlations between esds in cell parameters are only used when they are defined by crystal symmetry. An approximate (isotropic) treatment of cell esds is used for estimating esds involving l.s. planes.

### Fractional atomic coordinates and isotropic or equivalent isotropic displacement parameters (Å^2^)

**Table:**

	*x*	*y*	*z*	*U*_iso_*/*U*_eq_	
Br1	0.21338 (9)	1.56160 (8)	0.81718 (9)	0.0297 (3)	
Br2	−0.02997 (9)	1.55748 (8)	0.89049 (9)	0.0301 (3)	
Br3	0.03558 (8)	0.82412 (7)	0.99749 (8)	0.0252 (3)	
Br4	0.28295 (8)	0.82050 (7)	0.92686 (8)	0.0265 (3)	
N1	0.1827 (7)	1.3717 (6)	1.0200 (7)	0.0230 (19)	
H1	0.2019	1.3221	1.0555	0.028*	
N2	0.0484 (7)	1.2123 (6)	1.0839 (6)	0.0204 (18)	
H2	0.0886	1.2076	1.0402	0.024*	
N3	0.2148 (7)	1.0609 (6)	1.0661 (6)	0.0167 (16)	
H3	0.2260	1.1203	1.0869	0.020*	
N4	0.3381 (7)	1.2157 (6)	0.9795 (7)	0.0222 (19)	
H4	0.2697	1.2093	0.9724	0.027*	
CA1	0.2450 (8)	1.4179 (7)	0.9635 (8)	0.022 (2)	
CA2	0.0851 (8)	1.4144 (7)	1.0124 (8)	0.020 (2)	
CA3	0.0030 (8)	1.2964 (7)	1.1094 (8)	0.019 (2)	
CA4	0.0213 (8)	1.1366 (7)	1.1364 (7)	0.0183 (19)	
CA5	0.1262 (8)	1.0075 (7)	1.0743 (8)	0.0193 (19)	
CA6	0.2834 (8)	1.0081 (7)	1.0211 (7)	0.0178 (19)	
CA7	0.4088 (8)	1.1407 (7)	0.9972 (8)	0.022 (2)	
CA8	0.3906 (8)	1.3014 (7)	0.9749 (8)	0.021 (2)	
CB1	0.1808 (9)	1.4904 (8)	0.9173 (8)	0.025 (2)	
CB2	0.0831 (8)	1.4884 (7)	0.9456 (9)	0.023 (2)	
C11	−0.0765 (10)	1.4564 (8)	1.0813 (9)	0.027 (2)	
CB3	−0.0511 (8)	1.2742 (6)	1.1835 (8)	0.018 (2)	
HB3	−0.0902	1.3188	1.2147	0.021*	
C12	−0.0536 (10)	1.5524 (8)	1.1070 (9)	0.029 (3)	
H12	0.0171	1.5727	1.1175	0.035*	
CB4	−0.0378 (8)	1.1765 (7)	1.2033 (8)	0.020 (2)	
HB4	−0.0630	1.1425	1.2518	0.024*	
C13	−0.1339 (12)	1.6199 (9)	1.1176 (11)	0.038 (3)	
H13	−0.1173	1.6847	1.1358	0.045*	
CB5	0.1381 (7)	0.9188 (7)	1.0280 (7)	0.0163 (18)	
C14	−0.2362 (13)	1.5906 (11)	1.1012 (12)	0.044 (4)	
H14	−0.2905	1.6356	1.1082	0.052*	
CB6	0.2333 (9)	0.9172 (7)	0.9968 (8)	0.022 (2)	
C15	−0.2605 (11)	1.4969 (10)	1.0750 (11)	0.039 (3)	
H15	−0.3316	1.4778	1.0640	0.047*	
CB7	0.5103 (8)	1.1810 (7)	1.0048 (8)	0.019 (2)	
HB7	0.5738	1.1459	1.0165	0.023*	
C16	−0.1826 (10)	1.4296 (9)	1.0642 (10)	0.031 (3)	
H16	−0.2008	1.3653	1.0453	0.037*	
CB8	0.5011 (8)	1.2787 (8)	0.9925 (8)	0.023 (2)	
HB8	0.5569	1.3240	0.9949	0.028*	
CM1	0.0064 (9)	1.3882 (7)	1.0662 (8)	0.021 (2)	
CM2	0.0454 (8)	1.0384 (7)	1.1241 (7)	0.0180 (19)	
CM3	0.3822 (9)	1.0421 (8)	1.0052 (8)	0.023 (2)	
CM4	0.3474 (9)	1.3949 (7)	0.9573 (8)	0.022 (2)	
C21	−0.0149 (9)	0.9650 (7)	1.1696 (8)	0.023 (2)	
C22	−0.1224 (9)	0.9712 (8)	1.1722 (9)	0.026 (2)	
H22	−0.1601	1.0230	1.1416	0.031*	
C23	−0.1751 (10)	0.9040 (9)	1.2179 (10)	0.032 (3)	
H23	−0.2481	0.9103	1.2202	0.038*	
C24	−0.1207 (11)	0.8276 (8)	1.2602 (10)	0.031 (3)	
H24	−0.1567	0.7800	1.2909	0.038*	
C25	−0.0156 (12)	0.8192 (8)	1.2585 (10)	0.032 (3)	
H25	0.0203	0.7661	1.2887	0.039*	
C26	0.0404 (9)	0.8866 (7)	1.2135 (8)	0.022 (2)	
H26	0.1136	0.8800	1.2125	0.026*	
C31	0.4696 (9)	0.9730 (7)	0.9965 (8)	0.021 (2)	
C32	0.5005 (9)	0.9087 (7)	1.0679 (9)	0.026 (2)	
H32	0.4619	0.9049	1.1184	0.031*	
C33	0.5880 (9)	0.8506 (8)	1.0642 (9)	0.026 (2)	
H33	0.6103	0.8077	1.1126	0.031*	
C34	0.6427 (8)	0.8558 (8)	0.9893 (10)	0.028 (3)	
H34	0.7027	0.8163	0.9871	0.033*	
C35	0.6114 (9)	0.9172 (8)	0.9182 (10)	0.028 (3)	
H35	0.6485	0.9183	0.8666	0.034*	
C36	0.5244 (9)	0.9785 (7)	0.9218 (9)	0.024 (2)	
H36	0.5037	1.0226	0.8740	0.029*	
C41	0.4185 (9)	1.4724 (7)	0.9340 (9)	0.025 (2)	
C42	0.4811 (10)	1.4640 (8)	0.8651 (9)	0.028 (3)	
H42	0.4788	1.4062	0.8299	0.034*	
C43	0.5470 (10)	1.5388 (9)	0.8468 (10)	0.031 (3)	
H43	0.5915	1.5309	0.8007	0.038*	
C44	0.5490 (9)	1.6256 (8)	0.8951 (10)	0.030 (3)	
H44	0.5921	1.6778	0.8804	0.036*	
C45	0.4872 (11)	1.6347 (8)	0.9650 (11)	0.037 (3)	
H45	0.4896	1.6930	0.9994	0.044*	
C46	0.4211 (9)	1.5588 (7)	0.9858 (10)	0.030 (3)	
H46	0.3789	1.5654	1.0337	0.036*	
Cl1	0.0799 (2)	1.22478 (18)	0.8343 (2)	0.0237 (5)	
O11	0.1277 (7)	1.1716 (5)	0.9135 (6)	0.0240 (16)	
O12	0.1604 (8)	1.2791 (7)	0.7995 (8)	0.041 (2)	
O13	0.0358 (9)	1.1571 (7)	0.7677 (7)	0.045 (3)	
O14	0.0026 (8)	1.2868 (8)	0.8638 (8)	0.046 (3)	
Cl2	0.2813 (2)	1.26950 (19)	1.2481 (2)	0.0262 (6)	
O21	0.2935 (8)	1.2403 (7)	1.1561 (7)	0.036 (2)	
O22	0.3723 (8)	1.2469 (10)	1.3065 (9)	0.055 (3)	
O23	0.1930 (9)	1.2212 (9)	1.2770 (8)	0.050 (3)	
O24	0.2607 (13)	1.3723 (8)	1.2457 (11)	0.071 (4)	
Cl21	−0.2688 (4)	1.3297 (5)	0.6411 (4)	0.0755 (15)	
Cl22	−0.2737 (5)	1.3405 (5)	0.8337 (4)	0.0818 (17)	
C20	−0.201 (2)	1.357 (3)	0.7458 (17)	0.110 (11)	
H20A	−0.1377	1.3155	0.7555	0.132*	
H20B	−0.1771	1.4256	0.7455	0.132*	
Cl31	0.6956 (2)	1.2315 (2)	1.1840 (2)	0.0347 (7)	
Cl32	0.5739 (4)	1.0744 (3)	1.2483 (3)	0.0546 (11)	
C30	0.5833 (10)	1.2005 (9)	1.2365 (9)	0.030 (3)	
H30A	0.5874	1.2315	1.2968	0.037*	
H30B	0.5201	1.2252	1.1994	0.037*	
Cl41	−0.1911 (3)	1.0330 (4)	0.7629 (3)	0.0619 (12)	
Cl42	−0.1778 (4)	1.1044 (4)	0.9474 (4)	0.0691 (13)	
C40	−0.1286 (14)	1.0241 (13)	0.8713 (14)	0.056 (5)	
H40A	−0.0534	1.0373	0.8702	0.068*	
H40B	−0.1357	0.9567	0.8932	0.068*	

### Atomic displacement parameters (Å^2^)

**Table:**

	*U*^11^	*U*^22^	*U*^33^	*U*^12^	*U*^13^	*U*^23^
Br1	0.0310 (6)	0.0282 (5)	0.0307 (7)	0.0011 (4)	0.0075 (5)	0.0114 (5)
Br2	0.0270 (6)	0.0289 (5)	0.0346 (8)	0.0086 (4)	0.0039 (5)	0.0105 (5)
Br3	0.0235 (5)	0.0230 (5)	0.0293 (7)	−0.0065 (4)	0.0046 (5)	−0.0061 (4)
Br4	0.0252 (5)	0.0233 (5)	0.0317 (7)	−0.0004 (4)	0.0056 (5)	−0.0104 (4)
N1	0.025 (4)	0.016 (4)	0.027 (6)	0.000 (3)	−0.002 (4)	0.004 (3)
N2	0.026 (4)	0.020 (4)	0.018 (5)	0.004 (3)	0.012 (4)	0.002 (3)
N3	0.015 (4)	0.017 (3)	0.020 (5)	0.000 (3)	0.005 (3)	0.000 (3)
N4	0.020 (4)	0.021 (4)	0.025 (6)	0.000 (3)	0.002 (4)	0.004 (3)
CA1	0.016 (5)	0.017 (4)	0.033 (7)	−0.009 (3)	0.006 (4)	0.002 (4)
CA2	0.018 (5)	0.019 (4)	0.025 (6)	0.001 (3)	0.003 (4)	0.000 (4)
CA3	0.018 (4)	0.019 (4)	0.020 (6)	−0.002 (3)	0.006 (4)	−0.003 (4)
CA4	0.021 (5)	0.019 (4)	0.015 (6)	−0.002 (3)	0.003 (4)	0.002 (4)
CA5	0.020 (5)	0.022 (4)	0.016 (6)	0.002 (4)	0.001 (4)	−0.001 (4)
CA6	0.018 (4)	0.020 (4)	0.016 (6)	0.004 (3)	0.003 (4)	0.004 (4)
CA7	0.020 (5)	0.015 (4)	0.031 (7)	0.000 (3)	0.006 (4)	−0.003 (4)
CA8	0.018 (5)	0.017 (4)	0.027 (7)	−0.006 (3)	0.001 (4)	−0.004 (4)
CB1	0.032 (6)	0.025 (5)	0.019 (6)	−0.003 (4)	0.005 (5)	0.004 (4)
CB2	0.013 (4)	0.020 (4)	0.036 (7)	−0.003 (3)	0.005 (4)	0.008 (4)
C11	0.037 (6)	0.019 (5)	0.025 (7)	−0.001 (4)	0.006 (5)	−0.004 (4)
CB3	0.018 (4)	0.014 (4)	0.022 (6)	0.006 (3)	0.002 (4)	−0.002 (3)
C12	0.029 (6)	0.026 (5)	0.032 (8)	−0.007 (4)	0.005 (5)	−0.009 (5)
CB4	0.022 (5)	0.019 (5)	0.021 (6)	0.003 (3)	0.007 (4)	−0.003 (4)
C13	0.045 (7)	0.024 (5)	0.044 (9)	0.012 (5)	0.008 (6)	−0.013 (5)
CB5	0.015 (4)	0.017 (4)	0.017 (6)	−0.005 (3)	0.001 (4)	−0.003 (3)
C14	0.046 (8)	0.038 (7)	0.048 (10)	0.015 (6)	0.010 (7)	−0.005 (6)
CB6	0.027 (5)	0.014 (4)	0.026 (6)	0.004 (4)	0.006 (4)	0.006 (4)
C15	0.034 (7)	0.041 (7)	0.043 (9)	0.008 (5)	0.004 (6)	0.002 (6)
CB7	0.017 (5)	0.020 (4)	0.019 (6)	−0.001 (3)	−0.002 (4)	0.002 (4)
C16	0.027 (6)	0.035 (6)	0.029 (8)	0.002 (5)	0.001 (5)	−0.003 (5)
CB8	0.016 (5)	0.027 (5)	0.026 (7)	−0.003 (4)	0.003 (4)	0.005 (4)
CM1	0.028 (5)	0.019 (4)	0.016 (6)	−0.002 (4)	0.004 (4)	−0.003 (4)
CM2	0.022 (5)	0.022 (4)	0.010 (5)	−0.001 (4)	0.006 (4)	−0.001 (3)
CM3	0.023 (5)	0.025 (5)	0.022 (6)	−0.001 (4)	0.005 (4)	0.005 (4)
CM4	0.024 (5)	0.019 (4)	0.026 (7)	−0.001 (4)	0.010 (4)	−0.004 (4)
C21	0.022 (5)	0.023 (5)	0.025 (7)	−0.006 (4)	0.011 (4)	−0.005 (4)
C22	0.026 (6)	0.029 (5)	0.024 (7)	−0.007 (4)	0.005 (5)	−0.004 (4)
C23	0.021 (5)	0.042 (6)	0.033 (8)	−0.016 (5)	0.006 (5)	−0.007 (5)
C24	0.042 (7)	0.029 (6)	0.024 (7)	−0.013 (5)	0.006 (5)	0.002 (5)
C25	0.049 (8)	0.024 (5)	0.027 (8)	−0.014 (5)	0.015 (6)	0.001 (4)
C26	0.029 (5)	0.018 (4)	0.016 (6)	−0.003 (4)	−0.006 (4)	−0.002 (4)
C31	0.023 (5)	0.017 (4)	0.023 (6)	−0.005 (4)	−0.001 (4)	−0.005 (4)
C32	0.034 (6)	0.013 (4)	0.030 (7)	0.000 (4)	0.003 (5)	0.006 (4)
C33	0.026 (5)	0.019 (4)	0.033 (7)	0.002 (4)	0.004 (5)	0.003 (4)
C34	0.017 (5)	0.020 (5)	0.046 (8)	0.001 (4)	0.003 (5)	−0.006 (5)
C35	0.022 (5)	0.024 (5)	0.041 (8)	0.006 (4)	0.010 (5)	−0.009 (5)
C36	0.025 (5)	0.020 (4)	0.027 (7)	0.006 (4)	0.007 (4)	0.004 (4)
C41	0.022 (5)	0.020 (5)	0.032 (7)	0.004 (4)	0.004 (5)	0.006 (4)
C42	0.034 (6)	0.023 (5)	0.030 (7)	0.001 (4)	0.009 (5)	0.004 (4)
C43	0.025 (6)	0.038 (6)	0.032 (8)	−0.001 (5)	0.004 (5)	0.005 (5)
C44	0.029 (6)	0.023 (5)	0.036 (8)	−0.007 (4)	−0.011 (5)	0.013 (5)
C45	0.042 (7)	0.019 (5)	0.049 (9)	−0.012 (5)	0.005 (6)	−0.001 (5)
C46	0.025 (5)	0.018 (5)	0.047 (8)	−0.003 (4)	0.007 (5)	−0.002 (5)
Cl1	0.0233 (12)	0.0263 (11)	0.0214 (16)	0.0024 (9)	0.0024 (10)	−0.0023 (9)
O11	0.034 (4)	0.022 (4)	0.015 (4)	0.001 (3)	0.000 (3)	−0.002 (3)
O12	0.038 (5)	0.040 (5)	0.046 (7)	−0.005 (4)	0.014 (5)	0.014 (4)
O13	0.064 (7)	0.038 (5)	0.030 (6)	−0.007 (5)	−0.008 (5)	−0.012 (4)
O14	0.031 (5)	0.069 (7)	0.039 (7)	0.027 (5)	0.003 (4)	−0.006 (5)
Cl2	0.0264 (12)	0.0276 (12)	0.0252 (16)	−0.0027 (10)	0.0049 (11)	−0.0046 (10)
O21	0.049 (6)	0.033 (4)	0.027 (6)	−0.001 (4)	0.009 (4)	−0.001 (4)
O22	0.027 (5)	0.092 (9)	0.045 (8)	0.010 (5)	−0.003 (5)	−0.003 (6)
O23	0.038 (6)	0.067 (7)	0.046 (7)	−0.016 (5)	0.011 (5)	0.006 (5)
O24	0.111 (12)	0.032 (5)	0.075 (10)	0.011 (6)	0.039 (9)	−0.009 (6)
Cl21	0.055 (3)	0.124 (5)	0.046 (3)	−0.012 (3)	−0.002 (2)	0.004 (3)
Cl22	0.067 (3)	0.131 (5)	0.047 (3)	0.025 (3)	0.006 (2)	−0.015 (3)
C20	0.078 (17)	0.21 (3)	0.034 (15)	−0.017 (19)	−0.016 (11)	0.014 (17)
Cl31	0.0267 (14)	0.0446 (16)	0.0337 (19)	−0.0082 (12)	0.0071 (12)	−0.0090 (13)
Cl32	0.059 (2)	0.0410 (18)	0.068 (3)	−0.0209 (16)	0.024 (2)	−0.0156 (18)
C30	0.030 (6)	0.034 (6)	0.028 (7)	−0.005 (5)	0.009 (5)	−0.006 (5)
Cl41	0.036 (2)	0.105 (4)	0.044 (3)	0.005 (2)	−0.0038 (17)	−0.008 (2)
Cl42	0.070 (3)	0.087 (3)	0.049 (3)	−0.023 (3)	0.002 (2)	−0.008 (2)
C40	0.047 (9)	0.054 (9)	0.065 (13)	0.004 (7)	−0.009 (8)	0.012 (8)

### Geometric parameters (Å, º)

**Table:**

Br1—CB1	1.872 (11)	CM4—C41	1.475 (14)
Br2—CB2	1.851 (11)	C21—C22	1.395 (16)
Br3—CB5	1.874 (9)	C21—C26	1.411 (16)
Br4—CB6	1.849 (11)	C22—C23	1.373 (16)
N1—CA2	1.383 (13)	C22—H22	0.9500
N1—CA1	1.383 (13)	C23—C24	1.38 (2)
N1—H1	0.8807	C23—H23	0.9500
N2—CA3	1.369 (12)	C24—C25	1.36 (2)
N2—CA4	1.372 (13)	C24—H24	0.9500
N2—H2	0.8799	C25—C26	1.393 (15)
N3—CA5	1.377 (13)	C25—H25	0.9500
N3—CA6	1.377 (12)	C26—H26	0.9500
N3—H3	0.8800	C31—C36	1.386 (16)
N4—CA8	1.365 (12)	C31—C32	1.404 (16)
N4—CA7	1.382 (13)	C32—C33	1.389 (15)
N4—H4	0.8810	C32—H32	0.9500
CA1—CM4	1.371 (15)	C33—C34	1.387 (18)
CA1—CB1	1.422 (16)	C33—H33	0.9500
CA2—CM1	1.411 (15)	C34—C35	1.378 (19)
CA2—CB2	1.421 (15)	C34—H34	0.9500
CA3—CB3	1.405 (15)	C35—C36	1.410 (14)
CA3—CM1	1.420 (14)	C35—H35	0.9500
CA4—CM2	1.403 (14)	C36—H36	0.9500
CA4—CB4	1.432 (14)	C41—C42	1.382 (17)
CA5—CM2	1.413 (14)	C41—C46	1.415 (16)
CA5—CB5	1.419 (13)	C42—C43	1.382 (17)
CA6—CM3	1.403 (14)	C42—H42	0.9500
CA6—CB6	1.435 (15)	C43—C44	1.392 (19)
CA7—CM3	1.409 (14)	C43—H43	0.9500
CA7—CB7	1.414 (14)	C44—C45	1.39 (2)
CA8—CM4	1.414 (14)	C44—H44	0.9500
CA8—CB8	1.453 (15)	C45—C46	1.404 (15)
CB1—CB2	1.374 (15)	C45—H45	0.9500
C11—C12	1.397 (15)	C46—H46	0.9500
C11—C16	1.413 (18)	Cl1—O14	1.420 (9)
C11—CM1	1.460 (15)	Cl1—O12	1.425 (9)
CB3—CB4	1.382 (13)	Cl1—O13	1.429 (10)
CB3—HB3	0.9500	Cl1—O11	1.461 (9)
C12—C13	1.413 (17)	Cl2—O22	1.411 (12)
C12—H12	0.9500	Cl2—O23	1.428 (10)
CB4—HB4	0.9500	Cl2—O24	1.439 (11)
C13—C14	1.37 (2)	Cl2—O21	1.453 (10)
C13—H13	0.9500	Cl21—C20	1.74 (3)
CB5—CB6	1.363 (14)	Cl22—C20	1.71 (3)
C14—C15	1.37 (2)	C20—H20A	0.9900
C14—H14	0.9500	C20—H20B	0.9900
C15—C16	1.390 (18)	Cl31—C30	1.776 (12)
C15—H15	0.9500	Cl32—C30	1.749 (13)
CB7—CB8	1.361 (14)	C30—H30A	0.9900
CB7—HB7	0.9500	C30—H30B	0.9900
C16—H16	0.9500	Cl41—C40	1.72 (2)
CB8—HB8	0.9500	Cl42—C40	1.75 (2)
CM2—C21	1.485 (14)	C40—H40A	0.9900
CM3—C31	1.493 (15)	C40—H40B	0.9900
			
CA2—N1—CA1	110.1 (9)	CA1—CM4—CA8	124.0 (9)
CA2—N1—H1	124.6	CA1—CM4—C41	118.6 (9)
CA1—N1—H1	125.2	CA8—CM4—C41	117.4 (9)
CA3—N2—CA4	109.8 (8)	C22—C21—C26	119.0 (10)
CA3—N2—H2	125.0	C22—C21—CM2	123.1 (11)
CA4—N2—H2	125.1	C26—C21—CM2	117.9 (10)
CA5—N3—CA6	110.4 (8)	C23—C22—C21	121.6 (12)
CA5—N3—H3	124.7	C23—C22—H22	119.2
CA6—N3—H3	124.9	C21—C22—H22	119.2
CA8—N4—CA7	109.5 (9)	C22—C23—C24	119.0 (12)
CA8—N4—H4	125.2	C22—C23—H23	120.5
CA7—N4—H4	125.3	C24—C23—H23	120.5
CM4—CA1—N1	124.5 (10)	C25—C24—C23	120.8 (11)
CM4—CA1—CB1	129.9 (10)	C25—C24—H24	119.6
N1—CA1—CB1	105.5 (9)	C23—C24—H24	119.6
N1—CA2—CM1	123.6 (10)	C24—C25—C26	121.7 (13)
N1—CA2—CB2	107.7 (9)	C24—C25—H25	119.2
CM1—CA2—CB2	128.7 (10)	C26—C25—H25	119.2
N2—CA3—CB3	107.5 (8)	C25—C26—C21	117.9 (11)
N2—CA3—CM1	126.1 (9)	C25—C26—H26	121.0
CB3—CA3—CM1	126.3 (9)	C21—C26—H26	121.0
N2—CA4—CM2	125.7 (9)	C36—C31—C32	121.0 (10)
N2—CA4—CB4	107.1 (8)	C36—C31—CM3	119.7 (10)
CM2—CA4—CB4	127.2 (9)	C32—C31—CM3	119.2 (10)
N3—CA5—CM2	123.9 (9)	C33—C32—C31	119.5 (11)
N3—CA5—CB5	106.2 (8)	C33—C32—H32	120.2
CM2—CA5—CB5	129.9 (10)	C31—C32—H32	120.2
N3—CA6—CM3	123.6 (9)	C34—C33—C32	119.6 (11)
N3—CA6—CB6	106.6 (8)	C34—C33—H33	120.2
CM3—CA6—CB6	129.7 (9)	C32—C33—H33	120.2
N4—CA7—CM3	125.0 (10)	C35—C34—C33	121.2 (10)
N4—CA7—CB7	107.9 (8)	C35—C34—H34	119.4
CM3—CA7—CB7	127.1 (10)	C33—C34—H34	119.4
N4—CA8—CM4	127.4 (10)	C34—C35—C36	120.0 (11)
N4—CA8—CB8	106.7 (9)	C34—C35—H35	120.0
CM4—CA8—CB8	125.8 (9)	C36—C35—H35	120.0
CB2—CB1—CA1	110.1 (9)	C31—C36—C35	118.7 (11)
CB2—CB1—Br1	123.5 (9)	C31—C36—H36	120.6
CA1—CB1—Br1	125.4 (8)	C35—C36—H36	120.6
CB1—CB2—CA2	106.6 (9)	C42—C41—C46	119.7 (10)
CB1—CB2—Br2	123.9 (8)	C42—C41—CM4	123.2 (10)
CA2—CB2—Br2	128.8 (7)	C46—C41—CM4	117.1 (10)
C12—C11—C16	117.7 (11)	C41—C42—C43	120.7 (12)
C12—C11—CM1	121.0 (11)	C41—C42—H42	119.7
C16—C11—CM1	121.1 (10)	C43—C42—H42	119.7
CB4—CB3—CA3	108.5 (8)	C42—C43—C44	120.8 (12)
CB4—CB3—HB3	125.7	C42—C43—H43	119.6
CA3—CB3—HB3	125.7	C44—C43—H43	119.6
C11—C12—C13	121.2 (12)	C45—C44—C43	119.1 (11)
C11—C12—H12	119.4	C45—C44—H44	120.5
C13—C12—H12	119.4	C43—C44—H44	120.5
CB3—CB4—CA4	106.8 (9)	C44—C45—C46	120.9 (12)
CB3—CB4—HB4	126.6	C44—C45—H45	119.5
CA4—CB4—HB4	126.6	C46—C45—H45	119.5
C14—C13—C12	119.3 (12)	C45—C46—C41	118.8 (12)
C14—C13—H13	120.4	C45—C46—H46	120.6
C12—C13—H13	120.4	C41—C46—H46	120.6
CB6—CB5—CA5	109.4 (9)	O14—Cl1—O12	111.4 (7)
CB6—CB5—Br3	123.0 (8)	O14—Cl1—O13	111.6 (7)
CA5—CB5—Br3	127.1 (7)	O12—Cl1—O13	109.7 (7)
C15—C14—C13	120.6 (13)	O14—Cl1—O11	107.3 (6)
C15—C14—H14	119.7	O12—Cl1—O11	107.4 (6)
C13—C14—H14	119.7	O13—Cl1—O11	109.3 (5)
CB5—CB6—CA6	107.3 (9)	O22—Cl2—O23	110.4 (8)
CB5—CB6—Br4	126.0 (8)	O22—Cl2—O24	111.7 (9)
CA6—CB6—Br4	126.4 (8)	O23—Cl2—O24	108.3 (8)
C14—C15—C16	120.9 (14)	O22—Cl2—O21	110.2 (7)
C14—C15—H15	119.5	O23—Cl2—O21	109.3 (7)
C16—C15—H15	119.5	O24—Cl2—O21	106.7 (7)
CB8—CB7—CA7	108.1 (9)	Cl22—C20—Cl21	112.8 (16)
CB8—CB7—HB7	126.0	Cl22—C20—H20A	109.0
CA7—CB7—HB7	126.0	Cl21—C20—H20A	109.0
C15—C16—C11	120.3 (12)	Cl22—C20—H20B	109.0
C15—C16—H16	119.8	Cl21—C20—H20B	109.0
C11—C16—H16	119.8	H20A—C20—H20B	107.8
CB7—CB8—CA8	107.8 (9)	Cl32—C30—Cl31	110.6 (7)
CB7—CB8—HB8	126.1	Cl32—C30—H30A	109.5
CA8—CB8—HB8	126.1	Cl31—C30—H30A	109.5
CA2—CM1—CA3	123.1 (9)	Cl32—C30—H30B	109.5
CA2—CM1—C11	120.7 (9)	Cl31—C30—H30B	109.5
CA3—CM1—C11	116.2 (9)	H30A—C30—H30B	108.1
CA4—CM2—CA5	123.1 (9)	Cl41—C40—Cl42	113.2 (10)
CA4—CM2—C21	117.4 (9)	Cl41—C40—H40A	108.9
CA5—CM2—C21	119.5 (9)	Cl42—C40—H40A	108.9
CA6—CM3—CA7	124.8 (10)	Cl41—C40—H40B	108.9
CA6—CM3—C31	120.8 (9)	Cl42—C40—H40B	108.9
CA7—CM3—C31	114.4 (9)	H40A—C40—H40B	107.7
			
CA2—N1—CA1—CM4	177.8 (11)	CB3—CA3—CM1—C11	−19.5 (17)
CA2—N1—CA1—CB1	−1.8 (13)	C12—C11—CM1—CA2	−48.1 (18)
CA1—N1—CA2—CM1	−175.3 (11)	C16—C11—CM1—CA2	127.4 (14)
CA1—N1—CA2—CB2	2.5 (13)	C12—C11—CM1—CA3	131.2 (13)
CA4—N2—CA3—CB3	2.7 (13)	C16—C11—CM1—CA3	−53.3 (17)
CA4—N2—CA3—CM1	−174.8 (11)	N2—CA4—CM2—CA5	19.9 (18)
CA3—N2—CA4—CM2	173.8 (11)	CB4—CA4—CM2—CA5	−162.0 (12)
CA3—N2—CA4—CB4	−4.6 (13)	N2—CA4—CM2—C21	−162.9 (11)
CA6—N3—CA5—CM2	174.2 (10)	CB4—CA4—CM2—C21	15.1 (17)
CA6—N3—CA5—CB5	−3.0 (12)	N3—CA5—CM2—CA4	29.0 (17)
CA5—N3—CA6—CM3	−177.8 (10)	CB5—CA5—CM2—CA4	−154.4 (12)
CA5—N3—CA6—CB6	2.1 (12)	N3—CA5—CM2—C21	−148.1 (11)
CA8—N4—CA7—CM3	−179.1 (11)	CB5—CA5—CM2—C21	28.5 (18)
CA8—N4—CA7—CB7	0.5 (14)	N3—CA6—CM3—CA7	−27.5 (18)
CA7—N4—CA8—CM4	179.4 (12)	CB6—CA6—CM3—CA7	152.6 (13)
CA7—N4—CA8—CB8	−1.1 (14)	N3—CA6—CM3—C31	151.6 (11)
CM4—CA1—CB1—CB2	−179.2 (12)	CB6—CA6—CM3—C31	−28.2 (19)
N1—CA1—CB1—CB2	0.4 (13)	N4—CA7—CM3—CA6	−18 (2)
CM4—CA1—CB1—Br1	12.1 (19)	CB7—CA7—CM3—CA6	162.1 (12)
N1—CA1—CB1—Br1	−168.4 (8)	N4—CA7—CM3—C31	162.4 (11)
CA1—CB1—CB2—CA2	1.1 (14)	CB7—CA7—CM3—C31	−17.1 (18)
Br1—CB1—CB2—CA2	170.1 (9)	N1—CA1—CM4—CA8	28.0 (19)
CA1—CB1—CB2—Br2	−170.3 (9)	CB1—CA1—CM4—CA8	−152.5 (13)
Br1—CB1—CB2—Br2	−1.3 (15)	N1—CA1—CM4—C41	−149.8 (11)
N1—CA2—CB2—CB1	−2.2 (13)	CB1—CA1—CM4—C41	29.6 (19)
CM1—CA2—CB2—CB1	175.5 (12)	N4—CA8—CM4—CA1	17 (2)
N1—CA2—CB2—Br2	168.6 (9)	CB8—CA8—CM4—CA1	−162.9 (13)
CM1—CA2—CB2—Br2	−13.7 (19)	N4—CA8—CM4—C41	−165.5 (12)
N2—CA3—CB3—CB4	0.4 (13)	CB8—CA8—CM4—C41	15.0 (19)
CM1—CA3—CB3—CB4	177.9 (11)	CA4—CM2—C21—C22	42.4 (16)
C16—C11—C12—C13	1 (2)	CA5—CM2—C21—C22	−140.3 (12)
CM1—C11—C12—C13	177.1 (13)	CA4—CM2—C21—C26	−136.4 (11)
CA3—CB3—CB4—CA4	−3.2 (13)	CA5—CM2—C21—C26	40.9 (16)
N2—CA4—CB4—CB3	4.8 (12)	C26—C21—C22—C23	1.4 (18)
CM2—CA4—CB4—CB3	−173.6 (11)	CM2—C21—C22—C23	−177.4 (11)
C11—C12—C13—C14	−1 (2)	C21—C22—C23—C24	−1.7 (19)
N3—CA5—CB5—CB6	2.8 (12)	C22—C23—C24—C25	1 (2)
CM2—CA5—CB5—CB6	−174.2 (11)	C23—C24—C25—C26	−1 (2)
N3—CA5—CB5—Br3	−168.8 (8)	C24—C25—C26—C21	0.3 (19)
CM2—CA5—CB5—Br3	14.2 (18)	C22—C21—C26—C25	−0.7 (17)
C12—C13—C14—C15	0 (3)	CM2—C21—C26—C25	178.2 (11)
CA5—CB5—CB6—CA6	−1.6 (13)	CA6—CM3—C31—C36	127.8 (12)
Br3—CB5—CB6—CA6	170.4 (8)	CA7—CM3—C31—C36	−53.0 (15)
CA5—CB5—CB6—Br4	−176.5 (8)	CA6—CM3—C31—C32	−57.4 (16)
Br3—CB5—CB6—Br4	−4.4 (14)	CA7—CM3—C31—C32	121.8 (12)
N3—CA6—CB6—CB5	−0.3 (12)	C36—C31—C32—C33	0.8 (17)
CM3—CA6—CB6—CB5	179.6 (11)	CM3—C31—C32—C33	−173.9 (10)
N3—CA6—CB6—Br4	174.6 (8)	C31—C32—C33—C34	−1.1 (17)
CM3—CA6—CB6—Br4	−5.5 (18)	C32—C33—C34—C35	−0.3 (17)
C13—C14—C15—C16	0 (3)	C33—C34—C35—C36	2.0 (18)
N4—CA7—CB7—CB8	0.3 (14)	C32—C31—C36—C35	0.8 (17)
CM3—CA7—CB7—CB8	179.9 (12)	CM3—C31—C36—C35	175.5 (10)
C14—C15—C16—C11	1 (2)	C34—C35—C36—C31	−2.2 (17)
C12—C11—C16—C15	−1 (2)	CA1—CM4—C41—C42	−128.5 (13)
CM1—C11—C16—C15	−177.1 (13)	CA8—CM4—C41—C42	53.5 (18)
CA7—CB7—CB8—CA8	−1.0 (14)	CA1—CM4—C41—C46	51.5 (17)
N4—CA8—CB8—CB7	1.3 (14)	CA8—CM4—C41—C46	−126.5 (12)
CM4—CA8—CB8—CB7	−179.2 (11)	C46—C41—C42—C43	1 (2)
N1—CA2—CM1—CA3	−23.0 (18)	CM4—C41—C42—C43	−179.4 (12)
CB2—CA2—CM1—CA3	159.7 (12)	C41—C42—C43—C44	−2 (2)
N1—CA2—CM1—C11	156.2 (11)	C42—C43—C44—C45	3 (2)
CB2—CA2—CM1—C11	−21.1 (19)	C43—C44—C45—C46	−2 (2)
N2—CA3—CM1—CA2	−23.3 (19)	C44—C45—C46—C41	0 (2)
CB3—CA3—CM1—CA2	159.7 (11)	C42—C41—C46—C45	0.5 (19)
N2—CA3—CM1—C11	157.5 (11)	CM4—C41—C46—C45	−179.5 (12)

### Hydrogen-bond geometry (Å, º)

**Table:**

*D*—H···*A*	*D*—H	H···*A*	*D*···*A*	*D*—H···*A*
N1—H1···N2	0.88	2.57	3.018 (12)	113
N1—H1···O21	0.88	2.12	2.956 (14)	158
N2—H2···N1	0.88	2.60	3.018 (12)	110
N2—H2···N3	0.88	2.59	3.026 (12)	111
N2—H2···O11	0.88	2.07	2.896 (12)	157
N3—H3···O21	0.88	2.08	2.932 (13)	162
N4—H4···N3	0.88	2.62	3.034 (12)	110
N4—H4···O11	0.88	2.01	2.844 (13)	159
